# Heterogeneity of NK Cells and Other Innate Lymphoid Cells in Human and Murine Decidua

**DOI:** 10.3389/fimmu.2019.00170

**Published:** 2019-02-08

**Authors:** Paola Vacca, Laura Chiossone, Maria Cristina Mingari, Lorenzo Moretta

**Affiliations:** ^1^Department of Immunology, IRCCS Bambino Gesù Children's Hospital, Rome, Italy; ^2^Innate Pharma Research Labs, Innate Pharma, Marseille, France; ^3^Department of Experimental Medicine (DIMES) and Center of Excellence for Biomedical Research, University of Genoa, Genoa, Italy; ^4^UOC Immunology, IRCCS Ospedale Policlinico, San Martino, Genoa, Italy

**Keywords:** human and murine pregnancy, innate lymphoid cells (ILCs), natural killer (NK) cells, tolerance, decidua

## Abstract

Innate lymphoid cells (ILCs) represent a heterogeneous group of cells lacking genetically rearranged antigen receptors that derive from common lymphoid progenitors. Five major groups of ILCs have been defined based on their cytokine production pattern and developmental transcription factor requirements: namely, natural killer (NK) cells, ILC1s, ILC2s, ILC3s, and lymphoid tissue-inducer (LTi) cells. ILC1s, ILC2s, and ILC3s mirror the corresponding T helper subsets (Th1, Th2, and Th17, respectively) and produce cytokines involved in defense against pathogens, lymphoid organogenesis, and tissue remodeling. During the first trimester of pregnancy, decidual tissues contain high proportion of decidual NK (dNK) cells, representing up to 50% of decidual lymphocytes, and ILC3s. They release peculiar cytokines and chemokines that contribute to successful pregnancy. Recent studies revealed that ILCs display a high degree of plasticity allowing their prompt adaptation to environmental changes. Decidual NK cells may derive from peripheral blood NK cells migrated when pregnancy establishes or from *in situ* differentiation of hematopoietic precursors. Previous studies showed that human and murine decidua contain dNK cells, tissue resident NK cells, and ILC3s, all characterized by unique phenotypic and functional properties, most likely induced by decidual microenvironment to favor the establishment and the maintenance of pregnancy. Thus, during the early phase of pregnancy, the simultaneous presence of different ILC subsets further underscores the complexity of the cellular components of decidual tissues as well as the role of decidual microenvironment in shaping the plasticity and the function of ILCs.

## Introduction

**Figure 1:**
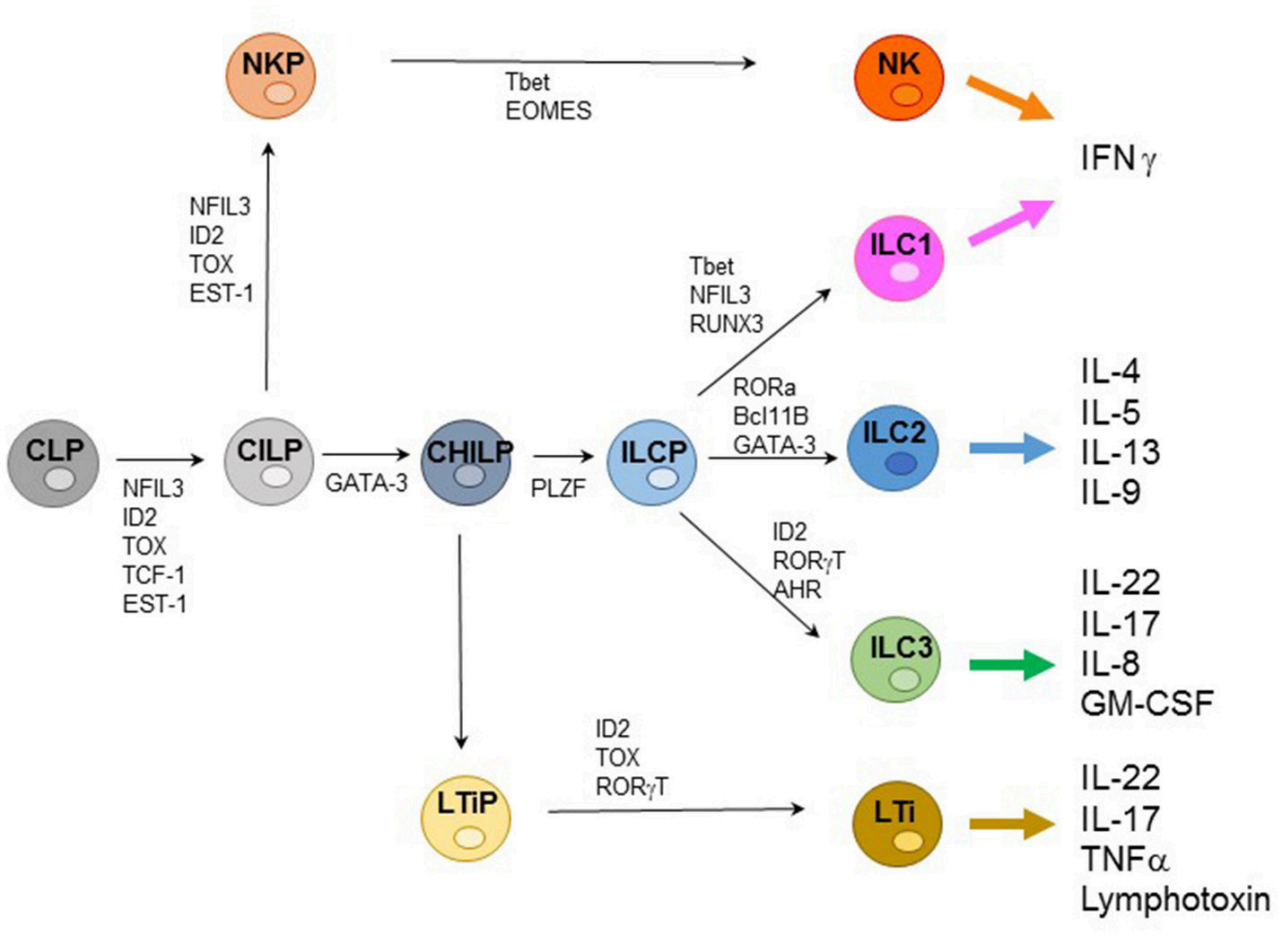
Representative ILC development and cytokine production. Common lymphoid progenitors (CLPs) differentiate into common innate lymphoid precursors (CILPs) that can further give rise to common helper innate lymphoid precursors (CHILPs) or into NK precursors (NKPs). CHILPs give rise to LTi precursors (LTiPs) that differentiate into LTi cells or to ILC precursors (ILCPs) that differentiate into ILC1s, ILC2s, and ILC3s, while NKPs differentiate toward NK cells. Specific transcription factors (TFs) required step are indicated.

ILCs represent an extended family of developmentally related hematopoietic cells that differ from T and B cells because they do not undergo somatic rearrangements of antigen-specific receptors. Notably, ILCs mirror the function of T cell subsets and contribute to host innate immune defenses, lymphoid organogenesis, and tissue remodeling. Based on their transcription factor (TF) profile ILCs have been recently classified in five groups including: (1) Natural Killer (NK cells); (2) ILC1s; (3) ILC2s; (4) ILC3s; and (5) Lymphoid Tissue-inducer cells (LTi) (1, 2). All ILCs derive from a common lymphoid progenitor (CLP) expressing the inhibitor of DNA binding 2 (ID2) TF. The CLPs differentiate into common innate lymphoid precursors (CILPs) that, in turn, can differentiate into common helper innate lymphoid precursors (CHILPs) or into NK precursors (NKPs). Moreover, CHILPs can subsequently give rise to LTi precursors (LTiPs) that differentiate into LTi cells or to ILC precursors (ILCPs) that give rise to ILC1s, ILC2s, and ILC3s, while NKPs differentiate toward NK cells. Notably each differentiation step is driven by specific TF (Figure 1) (3–5).

## Natural Killer Cells

NK cells display cytolytic activity against virus-infected and tumor cells and are characterized by the ability to rapidly release pro-inflammatory cytokines and chemokines involved in early inflammatory responses (6, 7). NK cell function is regulated by an array of inhibitory and activating receptors (8–13). Inhibitory NK receptors include Killer Ig-like Receptors (KIRs) in humans and Ly49 receptors in mice, that recognize classical MHC class-I molecules, and the heterodimer CD94/NKG2A able to interact with non-classical MHC class-I molecules (13, 14). Activating receptors include NKp46, NKG2D, and DNAM-1, in both humans and mice, and NKp30 and NKp44 that are expressed only by human NK cells. Other surface triggering molecules, such as 2B4 and NKp80, mainly function as co-receptors, enhancing natural cytotoxicity induced by triggering receptors. The most mature NK cells also express CD16, the low-affinity receptor for the Fc region of G-type immunoglobulins (IgG) responsible for antibody-dependent cell-mediated cytotoxicity (ADCC). In human, two NK cell subsets can be identified on the basis of CD16 and CD56 surface expression (15). CD56^dim^ NK cells, co-expressing CD16 and KIRs, are predominant in peripheral blood (PB), display potent cytolytic activity and rapidly release IFN-γ, whereas the poorly cytolytic CD56^bright^CD16^−^CD94/NKG2A^+^KIR^−^ NK cells are mostly found in tissues and secondary lymphoid organs where they are responsible for long-lasting production of chemokines and cytokines (16). Several studies demonstrated that NK cells, as other immune cells including ILCs, derive from a ID2^+^ hematopoietic precursor cell through the sequential acquisition of receptors and functions that allow the identification of distinct stages of development. Induction of NK cell commitment and further development require the expression of specific TFs as well as the exposure to a peculiar cytokine milieu. The TFs that drive NK cell differentiation are thymocyte selection-associated high mobility group box (TOX) and nuclear factor, interleukin 3 regulated (NFIL3, also known as E4BP4) (17). Achievement of later maturational stages also requires Eomesodermin (Eomes) and T-box transcription factor (Tbet) expression, which promotes the expression of cytolytic machinery and IFN-γ, respectively. Regarding the cytokine requirement, IL-15 is critical not only for the development of NK cells but also for their survival proliferation and function (2, 4, 5, 18). It is now clear that NK cell development not only occurs in the bone marrow (BM) but also in other peripheral lymphoid and non-lymphoid organs. Indeed, *ex vivo* maturational stages of NK cell differentiations have been identified in some tissues (e.g., thymus, tonsil, liver, and decidua) based on surface markers expression. In this context, NK cells have been extensively characterized in human and mouse decidual tissues.

**Figure 2:**
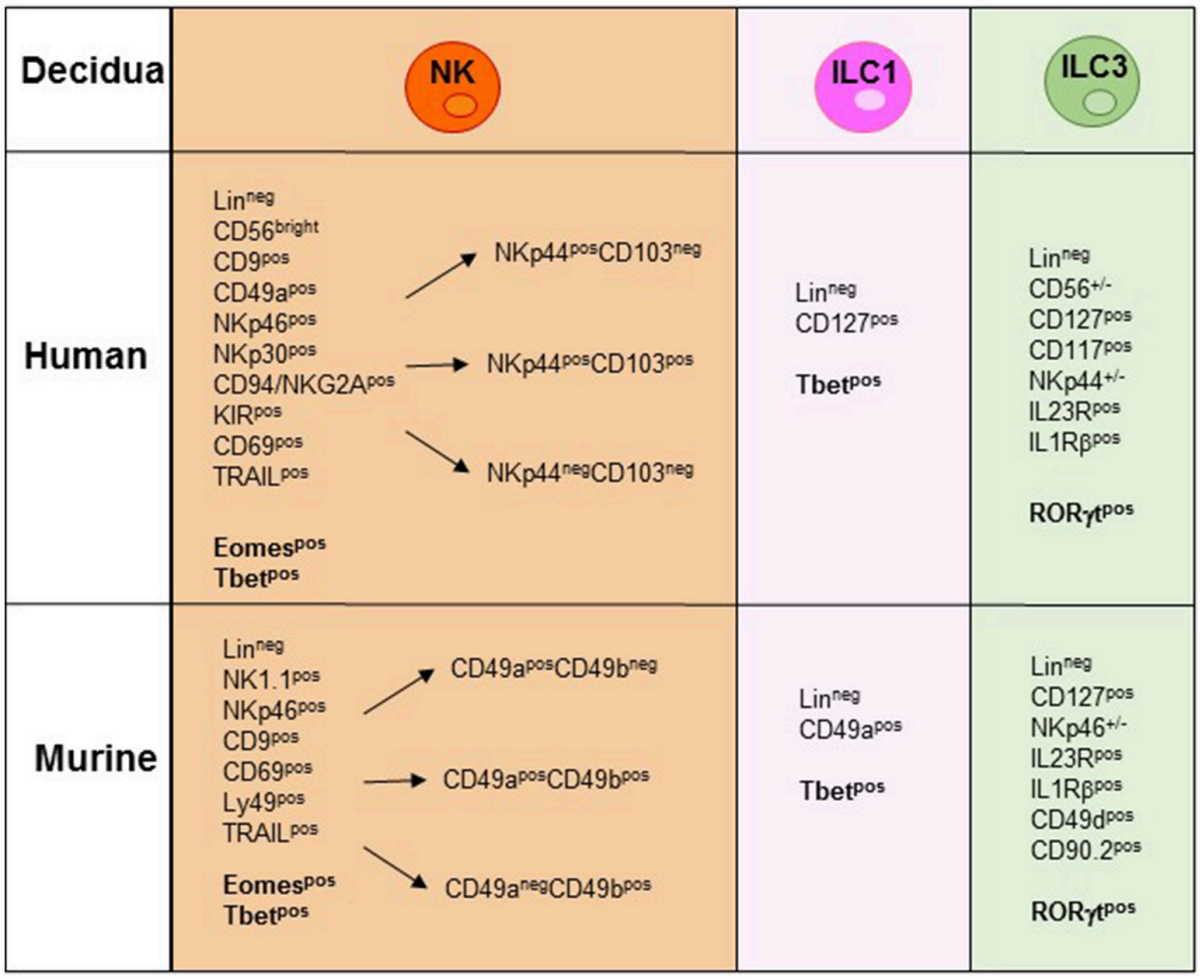
NK/ILC subsets present in human and murine decidua during the early phase of pregnancy. In the figure are indicated the surface markers and the transcription factors (TFs) expressed by the different human and murine NK/ILCs subsets. Lineage^neg^ (CD3^−^, CD19^−^, CD14^−^, CD123^−^, CD34^−^).

During the first trimester of pregnancy, NK cells reach 40–70% of total lymphocytes present in the decidua, representing the main lymphoid population and display unique phenotypic and functional features (19–23). Human decidua NK (dNK) cells are characterized by CD56^bright^CD16^−^KIR^+^CD9^+^CD49a^+^ phenotype, are poorly cytolytic and produce low amount of IFN-γ, as compared to PB-NK cells (24, 25) (Figure 2). Conversely, they secrete cytokines and chemokines e.g., VEGF, SDF-1, and IP-10 that promote neo-angiogenesis, tissue remodeling, immune modulation, and placentation (26–29). Moreover, dNK cells induce regulatory T cells (Tregs) that play a major role in the inhibition of maternal immune response and in tolerance induction (30, 31). In a recent paper, single-cell RNA sequencing of cells isolated from decidua and from the corresponding PB during the first trimester of pregnancy demonstrated the existence of three different NK cell subsets. These dNK subsets display a characteristic immunomodulatory profile and can specifically interact with other cells present in decidual microenvironment. The resulting cross-talk appears to play an important role in the control of successful pregnancy (32). It is of note that the microenvironment of different tumors displays an immunosuppressive milieu similar to that of decidua (33). Thus, a type of microenvironment playing a functional role in physiological condition, may instead favor tumor growth by suppressing the anti-tumor immune response. In particular, it has been shown that different types of cells present in the decidual microenvironment could exert a potent immunosuppressive activity inhibiting the function of NK cells (34–37). During murine gestation, metastatic spread is enhanced regardless of the tumor type and the decrease of NK cell activity is responsible of the observed increase in tumor metastases (33). It has been shown that human dNK cells express both inhibitory and activating KIRs specific for HLA-C molecules that are present at the trophoblast cell surface during the first trimester of pregnancy (30). Interactions occurring between KIRs and HLA-C molecules on trophoblast appear to play a relevant role in the induction of fetus-maternal tolerance (38, 39). In addition to KIRs, other receptors involved in the maintenance of pregnancy may be expressed by dNK cells. Of particular interest is NKG2C that upon binding to its corresponding ligand HLA-E, mediates the activation of NK cell function (23). In this context, the expression of NKG2C by dNK cells may play a key role in the control of cytomegalovirus (CMV) intrauterine infection during pregnancy (40). Notably, the frequency of NKG2C^+^ dNK cells increases during repeated pregnancies as compared to the first pregnancy. NKG2C^+^ dNK cell subset displays unique transcriptome and receptor profile and may sustain both vascularization and placentation during pregnancy (41). Recent studies provided evidence that NKG2C^+^ NK cells can specifically discriminate among different peptides bound to HLA-E. In particular, HLA-E-bound peptides derived from the leader sequence of HLA-G have been shown to induce an expansion of “adaptive” NK cells characterized by a high proliferative capacity and cytotoxicity (42, 43). Since HLA-G is mainly expressed by trophoblast cells it is possible to speculate that NKG2C and HLA-E binding to HLA-G peptides may play a relevant, still poorly explored, role in the maintenance of pregnancy.

The actual origin of dNK cells is not fully defined. Previous studies provided evidences that human decidual tissue contains CD34^+^ hematopoietic cell precursors expressing IL-15/IL-2 receptor β-chain, IL-7 receptor α-chain and mRNA encoding for E4BP4 and ID2 TF. Upon culture they could undergo *in vitro* differentiation into mature NK cells that display a phenotypical and functional profile similar to that of dNK cells. These observations strongly suggest that dCD34^+^ cells display a commitment to the NK cell lineage. Indeed, their differentiation occurs either in the presence of suitable growth factors or even upon co-culture with decidua-derived stromal cells strongly suggesting that dNK cells may derive from CD34^+^ precursors already present in the decidua (44). It has also been proposed that, since decidual microenvironment produces large amounts of attractant chemokines, dNK cells can also be recruited from periphery into decidual tissues when pregnancy establishes (45, 46). In particular, PB-NK cells migrating into decidua acquire both phenotypic and functional features typical of dNK cells thanks to the factors present in the local microenvironment (45, 47, 48). Notably, hematopoietic precursors are also found in decidua and uterus of pregnant mice. These precursors are committed to the NK cell lineage and undergo differentiation to NK cells in decidua and uterus during early pregnancy. In addition to precursors a large proportions of immature NK cells are found in decidua and uterus. These cells undergo rapid *in situ* proliferation/maturation. Immature murine dNK cells display low cytolytic activity and IFN-γ production. Moreover, dNK cells express high levels of Ly49 receptors, usually expressed by PB-NK cells. This resemble the expression of KIRs by human dNK cells (49). Moreover, it has been shown in mice that PB-NK cells display limited homing capacity to decidua and uterus, thus indicating that the recruitment from PB can only marginally contribute to the accumulation of NK cells in decidua and uterus. Thus, it is conceivable that the decidual microenvironment plays a key role in stimulating and supporting such rapid and unique NK cell differentiation (49). These observations allowed to identify decidua and uterus as novel sites for PB-NK cell differentiation as previously described for other peripheral sites (50). On the other hand, a recent study provided evidence that in mice the primary source of NK cells during pregnancy are tissue resident (tr) NK cells displaying a high proliferative capacity (51). Phenotypic and functional analysis of decidua and uterus-NK cells provided evidence of a previously unexpected high plasticity of NK cells. Indeed, the local microenvironment was found to shape the NK cell features during development and contribute to the acquisition of regulatory, rather than pro-inflammatory, function. These important correlations between mouse and human dNK cells may offer suitable tools for understanding the immune-regulation at the maternal-fetal interface and possibly, to clarify the pathogenesis of pregnancy-related diseases.

## Group 1 ILC

In addition to NK cells, ILC1s are another important source of IFNγ in peripheral tissues. However, ILC1s are more proficient in the production of TNF-α and, different from NK cells, they mainly reside within peripheral organs (52, 53). Whether ILC1s have also cytotoxic capabilities is currently unclear. While expressing very low levels of granzymes and perforin, they can induce TRAIL-mediated target cell killing. In addition, thanks to their ability to produce IFN-γ, ILC1s provide innate defenses against intracellular bacteria and protozoa (54, 55). The development of ILC1 depends on Tbet but not on Eomes, necessary for the development of mature NK cells. Although ILC1s express markers in common with NK cells and ILC3s (NK1.1 in mice and NKp44 and NKp46 in humans and mice, respectively), they can be identified thanks to the expression of CD127, CD49a, and TRAIL both in humans and mice. In humans, two subsets of ILC1 are described in the intestine: (1) NCR^−^Tbet^+^IFN-γ^+^ cells and (2) NKp44^+^CD103^+^ intraepithelial ILC1s (iILC1s) (56, 57). In particular, the first subset is characterized by high expression of CD127 and CD161 but lacks CD56, CD94, granzyme B and perforin (typical of mature NK cells). It express Tbet but not Eomes and reside in the lamina propria. The iILC1s share features in common with NK cells including the expression of CD56, the lack of CD127 and the localization in tonsils and in the intraepithelial space in the intestine. These cells are CD103^+^ and NKp44^+^ and express CD9 and CD49a, typical markers of dNK cells. Since CD103^+^ cells are Eomes^+^ and perforin^+^, it is possible that they represent a subset of NK cells rather than ILC1s.

In mice, Tbet^+^Eomes^+^ NK cells and Tbet^+^Eomes^−^ ILC1s represent two distinct lineages of differentiation, with Eomes^+^ NK cells originating from the BM and Tbet^+^ Eomes^−^ cells developing in peripheral organs (58). CD3^−^NK1.1^+^ cells characterized by Eomes^low/neg^ expression have been described in murine peripheral organs. In the liver, Eomes^low^ cells are found to be trNK cells characterized by a CD3^−^NK1.1^+^CD49a^+^DX5^−^ phenotype (53). However, the absence of Eomes expression, together with the presence of Tbet, rather suggests their belonging to the ILC1s (54). Notably, while in the liver the expression of CD49a is confined to Eomes^−^ cells, in decidua and uterus also a large proportion of Eomes^+^ cells are CD49a^+^, supporting the concept that CD49a expression alone does not allow discrimination between Eomes^+^NK cells and Eomes^−^ILC1 (58) (Figure 2). Previous studies in mice have shown that decidua and uterus NK cells express high levels of Eomes. Although NK1.1^+^Eomes^−^ ILC1s increased during pregnancy and specifically expanded in second pregnancies, Eomes^+^NK cells continued to represent the majority of uterine and decidual CD3^−^NK1.1^+^ cells. Importantly, both Eomes^+^ and Eomes^−^ subsets expressed Tbet. Moreover, based on CD49a, DX5 and Eomes expression, uterus and decidual cells could be further subdivided into different subsets, namely ILC1s (Eomes^−^CD49a^+^DX5^−^IFNγ^low^TNF^high^), common NK (cNK) cells (Eomes^+^CD49a^−^DX5^+^IFN-γ^high^TNF^low^), and two peculiar subsets of NK cells (Eomes^+^CD49a^+^DX5^−^IFNγ^+^TNF^+^ and Eomes^+^CD49a^+^DX5^+^IFN-γ^+^TNF^+^) that share phenotypic and functional features with cNK cells and the formerly described tissue resident NK (trNK) cells (53). A very recent study provided the first whole-genome transcriptome profile of the different ILC subsets present in decidua and uterus of mice during pregnancy. These results highlight the marked differences existing between the uterine resident CD49a^+^ trNK cells and the ILC1s (59). The abundance of Eomes^+^ cells in uterus and decidua suggests that they may derive from hematopoietic precursors of BM origin. However, as described above, dNK cells may also derive from accumulation of circulating immature cNK cells that upon exposure to tissue microenvironment acquire typical features of uterine NK cells including CD49a expression. Similarly, to mice, in human decidua, Eomes^+^ cells can be divided in three different subsets on the basis of NKp44 and CD103 expression. The CD103^+^ cells represent the major source of IFN-γ among dNK cells and may play a relevant role in the early inflammatory phase of pregnancy. Altogether, these studies indicate that the majority of ILCs present both in human and murine decidua are Eomes^+^ NK cells. Moreover, only in mice it is possible to identify also Eomes^−^ ILC1s (60–62). Notably, the decidual microenvironment may shape the conversion of a peculiar subset of ILC one into another. For example, in mice, TGF-β can induce the conversion of CD49a^−^CD49b^+^Eomes^+^NK cells into CD49a^+^CD49b^−^Eomes^low^ ILC1. Since this conversion may occur also in tumor microenvironment it may represent a further mechanism of tumor escape as ILC1 are characterized by reduced capacities to control tumor growth (63).

## Group 2 ILC

ILC2s have been originally identified in mice, they depend on GATA binding protein-3 (GATA3) TF for their development (64), and release IL-5, IL-9, IL-13, and small amounts of IL-4 in response to IL-25 and IL-33 stimulation. This cell subset plays an important role in the immune response against helminthic infections and is involved in allergic immune responses. In mice ILC2s are detectable in several tissues, including lymph nodes, fat-associated lymphoid clusters, spleen, liver, intestine, and airways while in humans are mainly found in lung and intestine (2). Studies in mice, demonstrated that ILC2s derive from an ID2^+^ precursor present in the BM and that their development is driven by RORα TF. ILC2s can be identified thanks to the expression of CRTH2, CD127, and CD25. They also express ICOS, which promotes ILC2 survival and cytokine production (65). They share with NK cells, ILC1s and ILC3s the expression of a number of activating and inhibitory receptors such as CD161, NKp30, KLRG1, and PD1 that can regulate their activation and function (66, 67).

The presence of ILC2s in decidual tissues is debated and may depend on the gestation phase. They are detectable in the uterine wall but not in decidua and endometrium both in humans and in mice. ILC2s have been detected in the uterus of both virgin and pregnant mice as well as in myometrium (Myo) and in mesometrial lymphoid aggregates (MLAp) (61, 62). Nfil3 TF is strictly required for the development and the expansion of uterine ILC2s in mice; indeed, in the uterus of virgin and pregnant Nfil^−/−^ mice ILC2s are not present. Importantly, thanks to their ability to release IL-5 in response to IL-25 and IL-33, ILC2s are involved in the control of the eosinophil homeostasis that, in turn, may play a role in the remodeling of uterine mucosa (68, 69). A study in humans provided evidence that ILC2s are abundant during preterm and term gestation at the fetal-maternal interface (70).

## Group 3 ILC

ILC3s represent a heterogeneous cell subset particularly abundant in mucosal tissues where they contribute to defenses against pathogens and to epithelial tissue homeostasis (1, 2, 71, 72). ILC3s are originally identified in the fetus and defined as LTi as they play a key role in driving lymphoid organogenesis. This capacity is partially related to the expression of lymphotoxin-alpha (LT-α) and LT-β that promote interactions with LTβ receptor (LTβ-R) expressing stromal cells. Upon engagement of LTβ-R, stromal cells upregulate adhesion molecules and secret chemokines that collectively promote the formation of lymph node anlagen (34, 73). Notably, cells with similar phenotypic characteristics have been identified also in adult secondary lymphoid organs and are defined as LTi-like cells. In the adult two subsets of ILC3s have recently been identified in mucosal tissues. They can be distinguished by the expression, or lack of NKp46 in mice and NKp44 in humans. ILC3s share common phenotypic features with both LTi-like cells and NK cells and express the RORγt TF, required for their differentiation and function. ILC3s, thanks to the ability to release IL-17 and IL-22, may contribute to host defenses by recruiting neutrophils and inducing the production of antimicrobial peptides (2, 4, 74, 75). Moreover, they are thought to induce tissue remodeling after acute inflammation (76). Although fetal LTi cells and adult ILC3s were previously considered to belong to the same ILC group, recent evidences revealed that they derive from two different precursors, namely, LTiP and ILCP, respectively, and follow separate developmental pathways.

Studies in mice demonstrated that, similarly to ILC2s, also ILC3s are present in virgin and pregnant uterus, in particular, in pregnant mice, they were enriched in Myo and MLAp but not in decidual tissues. During pregnancy, ILC3 numbers were higher than in virgin mice. Notably, the development of ILC3s was not dependent on the Nfil3 TF. However, in Nfil3^−/−^ mice ILC3 numbers were lower than in wild-type mice (61, 62).

In humans, ILC3s have been identified in decidual and endometrial tissues and include both NCR^+^ and NCR^−^ cell subsets (Figure 2). Decidual ILC3s express the hallmark of the ILC3 lineage, i.e., RORγt TF, CD127, CD117, IL-23R, and IL-1R. Human decidual NKp44^+^ILC3s not only produce IL-22, but are also the main source of IL-8 and GM-CSF while NCR^−^ILC3s mainly produce IL-17 and TNF-α (77, 78). These data are in line with those obtained in mice (49, 60). It is of note that a successful pregnancy requires an early inflammatory phase, necessary for implantation, while, at later stages a regulatory/immunosuppressive phase is needed to prevent fetal rejection (79). Since ILC3s release cytokines/chemokines involved in neutrophil recruitment/activation, neo-angiogenesis, tissue remodeling and placentation, they may actually play a key role not only in the early inflammatory phase but also in the induction of a tolerogenic status. Indeed, ILC3-derived IL-8 and GM-CSF are crucial for the recruitment of peripheral neutrophils into decidual tissues and for their activation and function. In turn, recruited neutrophils are necessary in the early inflammatory phase for a successful implantation. Thereafter, decidual neutrophils produce HB-EGF and IL1RA favoring the induction of tolerance (80). Moreover, decidual ILC3s interact with decidual stromal cells inducing the up-regulation of adhesion molecules on these cells. Notably, data on the role of ILC3s during pregnancy contributed to clarify the general involvement of these cells in tissues remodeling, inflammation and neo-angiogenesis. Regarding pregnancy, their effect on trophoblast invasion and placentation indicate that non-only dNK, but also ILC3s play a relevant role in the early phases of pregnancy (34, 35, 77).

## Conclusions

In this review, we recapitulate current knowledge on the presence and the role of NK cells and different ILCs in decidual tissues. Altogether, data highlight the complexity of uterine and decidual NK and ILC subsets. Such complexity, particularly during the first trimester of pregnancy, may reflect the effect of peculiar decidual microenvironment in shaping the features of both NK and ILC subsets. Although further analysis is clearly required to define their involvement in the establishment and maintenance of pregnancy, it is possible to speculate that a deficit of a peculiar NK or ILC subset or their altered function may result in pregnancy failure consequent to uncontrolled infection or deficient tissue and vessels formation.

## Author Contributions

All authors discussed together the general outline of the article. PV, LC, and LM wrote the first draft that was subsequently reviewed by MCM. Thereafter, all authors contributed to the elaboration of the final version of the manuscript.

### Conflict of Interest Statement

LC is employee of Innate Pharma. The remaining authors declare that the research was conducted in the absence of any commercial or financial relationships that could be construed as a potential conflict of interest.
